# Dimension reduction techniques for the integrative analysis of multi-omics data

**DOI:** 10.1093/bib/bbv108

**Published:** 2016-03-11

**Authors:** Chen Meng, Oana A. Zeleznik, Gerhard G. Thallinger, Bernhard Kuster, Amin M. Gholami, Aedín C. Culhane

**Keywords:** multivariate analysis, multi-omics data integration, dimension reduction, integrative genomics, exploratory data analysis, multi-assay

## Abstract

State-of-the-art next-generation sequencing, transcriptomics, proteomics and other high-throughput ‘omics' technologies enable the efficient generation of large experimental data sets. These data may yield unprecedented knowledge about molecular pathways in cells and their role in disease. Dimension reduction approaches have been widely used in exploratory analysis of single omics data sets. This review will focus on dimension reduction approaches for simultaneous exploratory analyses of multiple data sets. These methods extract the linear relationships that best explain the correlated structure across data sets, the variability both within and between variables (or observations) and may highlight data issues such as batch effects or outliers. We explore dimension reduction techniques as one of the emerging approaches for data integration, and how these can be applied to increase our understanding of biological systems in normal physiological function and disease.

## Introduction

Technological advances and lower costs have resulted in studies using multiple comprehensive molecular profiling or omics assays on each biological sample. Large national and international consortia including The Cancer Genome Atlas (TCGA) and The International Cancer Genome Consortium have profiled thousands of biological samples, assaying multiple different molecular profiles per sample, including mRNA, microRNA, methylation, DNA sequencing and proteomics. These data have the potential to reveal great insights into the mechanism of disease and to discover novel biomarkers; however, statistical methods for integrative analysis of multi-omics (or multi-assay) data are only emerging.

Exploratory data analysis (EDA) is an important early step in omics data analysis [1]. It summarizes the main characteristics of data and may identify potential issues such as batch effects [2] and outliers. Techniques for EDA include cluster analysis and dimension reduction. Both have been widely applied to transcriptomics data analysis [1], but there are advantages to dimension reduction approaches when integrating multi-assay data. While cluster analysis generally investigates pairwise distances between objects looking for fine relationships, dimension reduction or latent variable methods consider the global variance of the data set, highlighting general gradients or patterns in the data [3].

Biological data frequently have complex phenotypes and depending on the subset of variables analyzed, multiple valid clustering classifications may co-exist. Dimension reduction approaches decompose the data into a few new variables (called components) that explain most of the differences in observations. For example, a recent dimension reduction analysis of bladder cancers identified components associated with batch effects, GC content in the RNA sequencing data, in addition to seven components that were specific to tumor cells and three components associated with tumor stroma [4]. By contrast, most clustering approaches are optimized for discovery of discrete clusters, where each observation or variable is assigned to only one cluster. Limitations of clustering were observed when the method, cluster-of-cluster assignments, was applied to TCGA pan-cancer multi-omics data of 3527 specimens from 12 cancer type sources [5]. Tumors were assigned to one cluster, and these clusters grouped largely by anatomical origin and failed to identify clusters associated with known cancer pathways [5]. However, a dimension reduction analysis across 10 different cancers, identified novel and known cancer-specific pathways, in addition to pathways such as cell cycle, mitochondria, gender, interferon response and immune response that were common among different cancers [4].

Overlapping clusters have been identified in many tumors including glioblastoma and serous ovarian cancer [6, 7]. Gusenleitner and colleagues [8] found that k-means or hierarchical clustering failed to identify the correct cluster structure in simulated data with multiple overlapping clusters. Clustering methods may also falsely discover clusters in unimodal data. For example, Senbabaoğlu *et al.* [7] applied consensus clustering to randomly generated unimodal data and found it divided the data into apparently stable clusters for a range of K, where K is a predefined number of clusters. However, principal component analysis (PCA) did not identify these clusters.

In this article, we first introduce linear dimension reduction of a single data set, describing the fundamental concepts and terminology that are needed to understand its extensions to multiple matrices. Then we review multivariate dimension reduction approaches, which can be applied to the integrative exploratory analysis of multi-omics data. To demonstrate the application of these methods, we apply multiple co-inertia analysis (MCIA) to EDA of mRNA, miRNA and proteomics data of a subset of 60 cell lines studied at the National Cancer Institute (NCI-60).

## Introduction to dimension reduction

Dimension reduction methods arose in the early 20th century [9, 10] and have continued to evolve, often independently in multiple fields, giving rise to a myriad of associated terminology. Wikipedia lists over 10 different names for PCA, the most widely used dimension reduction approach. Therefore, we provide a glossary (Table 1) and tables of methods (Tables 2–4) to assist beginners to the field. Each of these are dimension reduction techniques, whether they are applied to one (Table 2) or multiple (Tables 3 and 4) data sets. We start by introducing the central concepts of dimension reduction.

**Table 1.** Glossary

Term	Definition
Variance	The variance of a random variable measures the spread (variability) of its realizations (values of the random variable). The variance is always a positive number. If the variance is small, the values of the random variable are close to the mean of the random variable (the spread of the data is low). A high variance is equivalent to widely spread values of the random variable. See [11].
Standard deviation	The standard deviation of a random variable measures the spread (variability) of its realizations (values of the random variable). It is defined as the square root of the variance. The standard deviation will have the same units as the random variable, in contrast to the variance. See [11].
Covariance	The covariance is an unstandardized measure about the tendency of two random variables to vary together. See [12].
Correlation	The correlation of two random variables is defined by the covariance of the two random variables normalized by the product between their standard deviations. It measures the linear relationship between the two random variables. The correlation coefficient ranges between −1 and +1. See [12].
Inertia	Inertia is a measure for the variability of the data. The inertia of a set of points relative to one point P is defined by the weighted sum of the squared distances between each considered point and the point P. Correspondingly, the inertia of a centered matrix (mean is equal to zero) is simply the sum of the squared matrix elements. The inertia of the matrix **X** defined by the metrics **L** and **D** is the weighted sum of its squared values. The inertia is equal the total variance of **X** when **X** is centered, **L** is the Euclidean metric and **D** is a diagonal matrix with diagonal elements equal to 1/*n*. See [13].
Co-inertia	The co-inertia is a global measure for the co-variability of two data sets (for example, two high-dimensional random variables). If the data sets are centered, the co-inertia is the sum of squared covariances. When coupling a pair of data sets, the co-inertia between two matrices, **X** and **Y**, is calculated as *trace* (**XLX**^T^**DYRY**^T^**D**). See [13].
Orthogonal	Two vectors are called orthogonal if they form an angle that measures 90 degrees. Generally, two vectors are orthogonal if their inner product is equal to zero. Two orthogonal vectors are always linearly independent. See [12].
Independent	In linear algebra, two vectors are called linearly independent if their liner combination is equal to zero only when all constants of the linear combination are equal to zero. See [14]. In statistics, two random variables are called statistically independent if the distribution of one of them does not affect the distribution of the other. If two independent random variables are added, then the mean of the sum is the sum of the two mean values. This is also true for the variance. The covariance of two independent variables is equal to zero. See [11].
Eigenvector, eigenvalue	An eigenvector of a matrix is a vector that does not change its direction after a linear transformation. The vector v is an eigenvector of the matrix **A** if: Av = λv. λ is the eigenvalue associated with the eigenvector v and it reflects the stretch of the eigenvector following the linear transformation. The most popular way to compute eigenvectors and eigenvalues is the SVD. See [14].
Linear combination	Mathematical expression calculated through the multiplication of variables with constants and adding the individual multiplication results. A linear combination of the variables *x* and *y* is ax + by where *a* and *b* are the constants. See [15].
Omics	The study of biological molecules in a comprehensive fashion. Examples of omics data types include genomics, transcriptomics, proteomics, metabolomics and epigenomics [16].
Dimension reduction	Dimension reduction is the mapping of data to a lower dimensional space such that redundant variance in the data is reduced or discarded, enabling a lower-dimensional representation without significant loss of information. See [17].
Exploratory data analysis	EDA is the application of statistical techniques that summarize the main characteristics of data, often with visual methods. In contrast to statistical hypothesis testing (confirmatory data analysis), EDA can help to generate hypotheses. See [18].
Sparse vector	A sparse vector is a vector in which most elements are zero. A sparse loadings matrix in PCA or related methods reduce the number of features contributing to a PC. The variables with nonzero entries (features) are the ‘selected features'. See [19].

**Table 2.** Dimension reduction methods for one data set

Method	Description	Name of R function {R package}
PCA	Principal component analysis	prcomp{stats}, princomp{stats}, dudi.pca{ade4}, pca{vegan}, PCA{FactoMineR}, principal{psych}
CA, COA	Correspondence analysis	ca{ca}, CA{FactoMineR}, dudi.coa{ade4}
NSC	Nonsymmetric correspondence analysis	dudi.nsc{ade4}
PCoA, MDS	Principal co-ordinate analysis/multiple dimensional scaling	cmdscale{stats} dudi.pco{ade4} pcoa{ape}
NMF	Nonnegative matrix factorization	nmf{nmf}
nmMDS	Nonmetric multidimensional scaling	metaMDS{vegan}
sPCA, nsPCA, pPCA	Sparse PCA, nonnegative sparse PCA, penalized PCA. (PCA with feature selection)	SPC{PMA}, spca{mixOmics}, nsprcomp{nsprcomp}, PMD{PMA}
NIPALS PCA	Nonlinear iterative partial least squares analysis (PCA on data with missing values)	nipals{ade4} pca{pcaMethods}^a^ nipals{mixOmics}
pPCA, bPCA	Probabilistic PCA, Bayesian PCA	pca{pcaMethods}^a^
MCA	Multiple correspondence analysis	dudi.acm{ade4}, mca{MASS}
ICA	Independent component analysis	fastICA{FastICA}
sIPCA	Sparse independent PCA (combines sPCA and ICA)	sipca{mixOmics} ipca{mixOmics}
plots	Graphical resources	R packages including scatterplot3d, ggord^b^, ggbiplot^c^, plotly^d^, explor

^a^Available in Bioconductor.

^b^On github: devtools::install_github (‘fawda123/ggord').

^c^On github: devtools::install_github (‘ggbiplot', ‘vqv').

^d^On github: devtools::install_github (‘ropensci/plotly').

**Table 3.** Dimension reduction methods for pairs of data sets

Method	Description	Feature selection	R Function {package}
CCA^a^	Canonical correlation analysis. Limited to *n* > p^a^	No	cc{cca} CCorA{vegan},
CCA^a^	Canonical correspondence analysis is a constrained correspondence analysis, which is popular in ecology^a^	No	cca{ade4} cca{vegan} cancor{stats}
RDA	Redundancy analysis is a constrained PCA. Popular in ecology	No	rda{vegan}
Procrutes	Procrutes rotation rotates a matrix to maximum similarity with a target matrix minimizing sum of squared differences	No	procrustes{vegan} procuste{ade4}
rCCA	Regularized canonical correlation	No	rcc{cca}
sCCA	Sparse CCA	Yes	CCA{pma}
pCCA	Penalized CCA	Yes	spCCA{spCCA} supervised version
WAPLS	Weighted averaging PLS regression	No	WAPLS{rioja}, wapls{paltran}
PLS	Partial least squares of K-tables (multi-block PLS)	No	mbpls{ade4}, plsda{caret}
sPLS pPLS	Sparse PLS Penalized PLS	Yes	spls{spls} spls{mixOmics} ppls{ppls}
sPLS-DA	Sparse PLS-discriminant analysis	Yes	splsda{mixOmics}, splsda{caret}
cPCA	Consensus PCA	No	cpca{mogsa}
CIA	Coinertia analysis	No	coinertia{ade4} cia{made4}

^a^A source for confusion, CCA is widely used as an acronym for both Canonical ‘Correspondence' Analysis and Canonical ‘Correlation' Analysis. Throughout this article we use CCA for canonical ‘correlation' analysis. Both methods search for the multivariate relationships between two data sets. Canonical ‘correspondence' analysis is an extension and constrained form of ‘correspondence' analysis [22]. Both canonical ‘correlation' analysis and RDA assume a linear model; however, RDA is a constrained PCA (and assumes one matrix is the dependent variable and one independent), whereas canonical correlation analysis considers both equally. See [23] for more explanation.

**Table 4.** Dimension reduction methods for multiple (more than two) data sets

Method	Description	Feature selection	Matched cases	R Function {package}
MCIA	Multiple coinertia analysis	No	No	mcia{omicade4}, mcoa{ade4}
gCCA	Generalized CCA	No	No	regCCA{dmt}
rGCCA	Regularized generalized CCA	No	No	regCCA{dmt} rgcca{rgcca} wrapper.rgcca{mixOmics}
sGCCA	Sparse generalized canonical correlation analysis	Yes	No	sgcca{rgcca} wrapper.sgcca{mixOmics}
STATIS	Structuration des Tableaux á Trois Indices de la Statistique (STATIS). Family of methods which include X-statis	No	No	statis{ade4}
CANDECOMP/ PARAFAC / Tucker3	Higher order generalizations of SVD and PCA. Require matched variables and cases.	No	Yes	CP{ThreeWay}, T3{ThreeWay}, PCAn{PTaK}, CANDPARA{PTaK}
PTA	Partial triadic analysis	No	Yes	pta{ade4},
statico	Statis and CIA (find structure between two pairs of K-tables)	No	No	statico{ade4}

We denote matrices with boldface uppercase letters. The rows of a matrix contain the observations, while the columns hold the variables. In an omics study, the variables (also referred to as features) generally measure tissue or cell attributes including abundance of mRNAs, proteins and metabolites. All vectors are columns vectors and are denoted with boldface lowercase letters. Scalars are indicated by italic letters.

Given an omics data set, **X**, which is an* n×p* matrix, of* n* observations and *p* variables, it can be represented by:

(1) $$\text{X}=\left({\text{x}}_{1},{\text{x}}_{2},...,{\text{x}}_{p}\right)$$

where **x** are vectors of length *n*, and are measurements of mRNA or other biological variables for *n* observations (samples). In a typical omics study, *p* ranges from several hundred to millions. Therefore, observations (samples) are represented in large dimensional spaces ℝ^p^. The goal of dimension reduction is to identify a (set of) new variable(s) using a linear combination of the original variables, such that the number of new variables is much smaller than *p*. An example of such a linear combination is shown in Equation (2);

(2) $$f=q_{1}x_{1}+q_{2}x_{2}+\ldots +q_{p}x_{p}$$

or expressed in a matrix form:

(3) f=Xq

In Equations (2) and (3), **f** is a new variable, which is often called a latent variable or a component. Depending on the scientific field, **f** may also be called principal axis, eigenvector or latent factor. **q**=(*q_1_*, *q_2_*,…, *q_p_*)^T^ is a *p*-length vector of coefficients of scalar values in which at least one of the coefficients is different from zero. These coefficients are also called ‘loadings'. Dimension reduction analysis introduces constraints to obtain a meaningful solution; we find the set of **q**’s that maximize the variance of components **f**’s. In doing so, a smaller number of variables, **f**, capture most of the variance in the data. Different optimization and constraint criteria distinguish between different dimension reduction methods. Table 2 provides a nonexhaustive list of these methods, which includes PCA, linear discriminant analysis and factor analysis.

## Principal component analysis

PCA is one of the most widely used dimension reduction methods [20]. Given a column centered and scaled (unit variance) matrix **X**, PCA finds a set of new variables **f**^i^=**Xq**^i^ where *i* is the *i*th component and **q**^i^ is the variable loading for the *i*th principal component (PC; superscript denotes the component or the dimension). The variance of **f**^i^ is maximized, that is:

(4) $${\text{arg max}}_{q^{i}}\text{ var(}Xq^{i})$$

with the constraints that ||**q**^i^|| = 1 and each pair of components (**f**^i^,**f**^j^) are orthogonal to each other (or uncorrelated, i.e. **f**^iT^**f**^j^ = 0 for j ≠ i).

PCA can be computed using different algorithms including eigen analysis, latent variable analysis, factor analysis, singular value decomposition (SVD) [21] or linear regression [3]. Among them, SVD is the most widely used approach. Given **X**, an *n×p* matrix, with rank r (r  min[n, p]), SVD decomposes **X** into three matrices:

(5) $$\text{X}={\text{USQ}}^{\text{T}}{\mathrm{subject to the constraint that}\text{U}}^{\text{T}}\text{U}={\text{Q}}^{T}\text{Q}=\text{I}$$

where **U** is an *n×r *matrix and **Q** is a *p×r* matrix. The columns of **U** and **Q** are the orthogonal left and right singular vectors, respectively. **S** is an *r×r *diagonal matrix of singular values, which are proportional to the standard deviations associated with *r *singular vectors. The singular vectors are ordered such that their associated variances are monotonically decreasing. In a PCA of **X**, the PCs comprise an *n×r* matrix, **F**, which is defined as:

(6) $$\text{F}=\text{US}={\text{USQ}}^{\text{T}}\text{Q}=\text{XQ}$$

where the columns of matrix **F** are the PCs and the matrix **Q** is called the loadings matrix and contains the linear combination coefficients of the variables for each PC (**q** in Equation (3)). Therefore, we represent the variance of **X** in lower dimension *r*. The above formula also emphasizes that **Q** is a matrix that projects the observations in **X** onto the PCs. The sum of squared values of the columns in **U** equals 1 (Equation (5)), therefore, the variance of the *i*th PC, $d^{i^{2}}$, can be calculated as

(7) $$d^{i^{2}}=\frac{s^{i^{2}}}{n-1}$$

where *s^i^* is the *i*th diagonal element in **S**. The variance reflects the amount of information (underling structure) captured by each PC. The squared correlations between PCs and the original variables are informative and often illustrated using a correlation circle plot. These can be calculated by:

(8) C=QD 

where **D** = (d^1^, d^2^,…, d^r^)^T^ is a diagonal matrix of the standard deviation of the PCs.

In contrast to SVD, which calculates all PCs simultaneously, PCA can also be calculated using the Nonlinear Iterative Partial Least Squares (NIPALS) algorithm, which uses an iterative regression procedure to calculate PCs. Computation can be performed on data with missing values and it is faster than SVD when applied to large matrices. Furthermore, NIPALS may be generalized to discover the correlated structure in more than one data set (see sections on the analysis of multi-omics data sets). Please refer to the Supplementary Information for additional details on NIPALS.

## Visualizing and interpreting results of dimension reduction analysis

**Figure 1.**
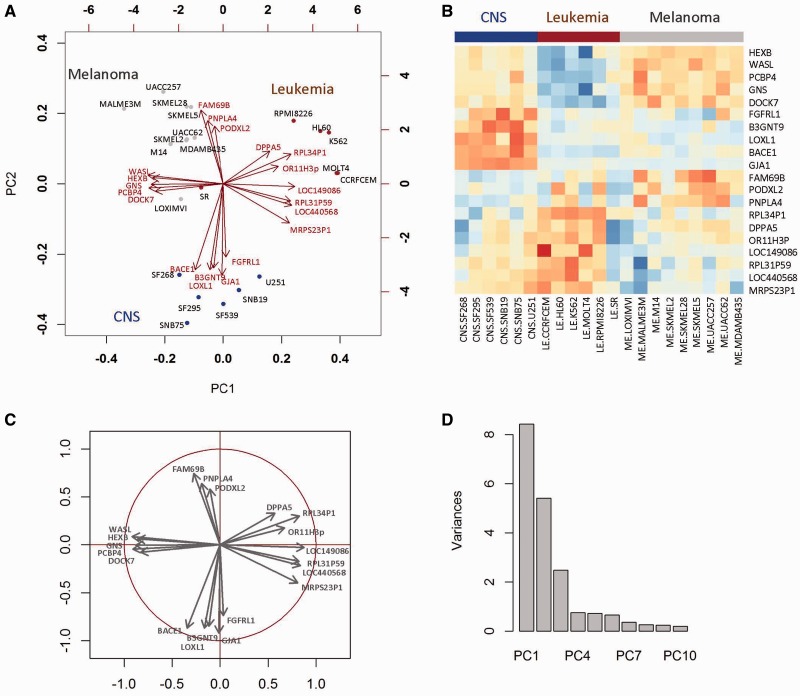
Results of a PCA analysis of mRNA gene expression data of melanoma (ME), leukemia (LE) and central nervous system (CNS) cell lines from the NCI-60 cell line panel. All variables were centered and scaled. Results show (**A**) a biplot where observations (cell lines) are points and gene expression profiles are arrows; (**B**) a heatmap showing the gene expression of the same 20 genes in the cell lines; red to blue scale represent high to low gene expression (light to dark gray represent high to low gene expression on the black and white figure); (**C**) correlation circle; (**D**) variance barplot of the first ten PCs. To improve the readability of the biplot, some labels of the variables (genes) in (**A**) have been moved slightly. A colour version of this figure is available online at BIB online: http://bib.oxfordjournals.org.

We present an example to illustrate how to interpret results of a PCA. PCA was applied to analyze mRNA gene expression data of a subset of cell lines from the NCI-60 panel; those of melanoma, leukemia and central nervous system (CNS) tumors. The results of PCA can be easily interpreted by visualizing the observations and variables in the same space using a biplot. Figure 1A is a biplot of the first (PC1) and second PC (PC2), where points and arrows from the plot origin, represent observations and genes, respectively. Cell lines (points) with correlated gene expression profiles have similar scores and are projected close to each other on PC1 and PC2. We see that cell lines from the same anatomical location are clustered.

Both the direction and length of the mRNA gene expression vectors can be interpreted. Gene expression vectors point in the direction of the latent variable (PC) to which it is most similar (squared correlation). Gene vectors with the same direction (e.g. FAM69B, PNPLA2, PODXL2) have similar gene expression profiles. The length of the gene expression vector is proportional to the squared multiple correlation between the fitted values for the variable and the variable itself.

A gene expression vector and a cell line projected in the same direction from the origin are positively associated. For example in Figure 1A, FAM69B, PNPLA2, PODXL2 are active (have higher gene expression) in melanoma cell lines. Similarly, genes DPPA5 and RPL34P1 are among those genes that are highly expressed in most leukemia cell lines. By contrast, genes WASL and HEXB point in the opposite direction to most leukemia cell lines indicating low association. In Figure 1B it is clear that these genes are not expressed in leukemia cell lines and are colored blue in the heatmap (these are colored dark gray in grayscale).

The sum of the squared correlation coefficients between a variable and all the components (calculated in equation 8) equals 1. Therefore, variables are often shown within a correlation circle (Figure 1C). Variables positioned on the unit circle represent variables that are perfectly represented by the two dimensions displayed. Those not on the unit circle may require additional components to be represented.

In most analyses, only the first few PCs are plotted and studied, as these explain the most variant trends in the data. Generally, the selection of components is subjective and depends on the purpose of the EDA. An informal elbow test may help to determine the number of PCs to retain and examine [24, 25]. From the scree plot of PC eigenvalues in Figure 1D, we might decide to examine the first three PCs because the decrease in PC variance becomes relatively moderate after PC3. Another approach that is widely used is to include (or retain) PCs that cumulatively capture a certain proportion of variance; for example, 70% of variance is modeled with three PCs. If a parsimony model is preferred, the variance proportion cutoff can be as low as 50% [24]. More formal tests, including the Q^2^ statistic, are also available (for details, see [24]). In practice, the first component might explain most of the variance and the remaining axes may simply be attributed to noise from technical or biological sources in a study with low complexity (e.g. cell line replicates of controls and one treatment condition). However, a complex data set (for example, a set of heterogeneous tumors) may require multiple PCs to capture the same amount of variance.

## Different dimension reduction approaches are optimized for different data

There are many dimension reduction approaches related to PCA (Table 2), including principal co-ordinate analysis (PCoA), correspondence analysis (CA) and nonsymmetrical correspondence analysis (NSCA). These may be computed by SVD, but differ in how the data are transformed before decomposition [21, 26, 27], and therefore, each is optimized for specific data properties. PCoA (also known as Classical Multidimensional Scaling) is versatile, as it is a SVD of a distance matrix that can be applied to decompose distance matrices of binary, count or continuous data. It is frequently applied in the analysis of microbiome data [28].

PCA is designed for the analysis of multi-normal distributed data. If data are strongly skewed or extreme outliers are present, the first few axes may only separate those objects with extreme values instead of displaying the main axes of variation. If data are unimodal or display nonlinear trends, one may see distortions or artifacts in the resulting plots, in which the second axis is an arched function of the first axis. In PCA, this is called the horseshoe effect and it is well described, including illustrations, in Legendre and Legendre [3]. Both nonmetric Multi-Dimensional Scaling (MDS) and CA perform better than PCA in these cases [26, 29]. Unlike PCA, CA can be applied to sparse count data with many zeros. Although designed for contingency tables of nonnegative count data, CA and NSCA, decompose a chi-squared matrix [30, 31], but have been successfully applied to continuous data including gene expression and protein profiles [32, 33]. As described by Fellenberg *et al.* [33], gene and protein expression can be seen as an approximation of the number of corresponding molecules present in the cell during a certain measured condition. Additionally, Greenacre [27] emphasized that the descriptive nature of CA and NSCA allows their application on data tables in general, not only on count data. These two arguments support the suitability of CA and NSCA as analysis methods for omics data. While CA investigates symmetric associations between two variables, NSCA captures asymmetric relations between variables. Spectral map analysis is related to CA, and performs comparably with CA, each outperforming PCA in the identification of clusters of leukemia gene expression profiles [26]. All dimension reduction methods can be formulated in terms of the duality diagram. Details on this powerful framework are included in the Supplementary Information.

Nonnegative matrix factorization (NMF) [34] forces a positive or nonnegative constraint on the resulting data matrices and, similar to Independent Component Analysis (ICA) [35], there is no requirement for orthogonality or independence in the components. The nonnegative constraint guarantees that only the additive combinations of latent variables are allowed. This may be more intuitive in biology where many biological measurements (e.g. protein concentrations, count data) are represented by positive values. NMF is described in more detail in the Supplemental Information. ICA was recently applied to molecular subtype discovery in bladder cancer [4]. Biton *et al.* [4] applied ICA to gene expression data of 198 bladder cancers and examined 20 components. ICA successfully decomposed and extracted multiple layers of signal from the data. The first two components were associated with batch effects but other components revealed new biology about tumor cells and the tumor microenvironment. They also applied ICA to non-bladder cancers and, by comparing the correlation between components, were able to identify a set of bladder cancer-specific components and their associated genes.

As omics data sets tend to have high dimensionality (*p* ≫ n) it is often useful to reduce the number of variables. Several recent extensions of PCA include variable selection, often via a regularization step or L-1 penalization (e.g. Least Absolute Shrinkage and Selection Operator, LASSO) [36]. The NIPALS algorithm uses an iterative regression approach to calculate the components and loadings, which is easily extended to have a sparse operator that can be included during regression on the component [37]. A cross-validation approach can be used to determine the level of sparsity. Sparse, penalized and regularized extensions of PCA and related methods have been described recently [36, 38–41].

## Integrative analysis of two data sets

One-table dimension reduction methods have been extended to the EDA of two matrices and can simultaneously decompose and integrate a pair of matrices that measure different variables on the same observations (Table 3). Methods include generalized SVD [42], Co-Inertia Analysis (CIA) [43, 44], sparse or penalized extensions of Partial Least Squares (PLS), Canonical Correspondence analysis (CCA) and Canonical Correlation Analysis (CCA) [36, 45–47]. Note both canonical correspondence analysis and canonical correlation analysis are referred to by the acronym CCA. Canonical correspondence analysis is a constrained form of CA that is widely used in ecological statistics [46]; however, it is yet to be adopted by the genomics community in analysis of pairs of omics data. By contrast, several groups have applied extensions of canonical correlation analysis to omics data integration. Therefore, in this review, we use CCA to describe canonical correlation analysis.

## Canonical correlation analysis

Two omics data sets **X** (dimension *n×p_x_*) and **Y** (dimension *n×p_y_*) can be expressed by the following latent component decomposition problem:

(9) $$\begin{array}{l} X\;=\;F_{x}Q_{x}^{T}+E_{x}\\ Y\;\text{= }F_{y}Q_{y}^{T}\;+\;E_{y} \end{array}$$

where **F_x_** and **F_y_** are *n×r* matrices, with *r* columns of components that explain the co-structure between **X** and **Y**. The columns of **Q_x_** and **Q_y_** are variable loading vectors for **X** and **Y**, respectively. **E_x_** and **E_y_** are error terms.

Proposed by Hotelling in 1936 [47], CCA searches for associations or correlations among the variables of **X** and **Y** [47], by maximizing the correlation between **Xq_x_**^i^ and** Yq_y_**^i^:

(10) $$\text{for the}i\text{th component}:\arg\;\max_{q_{x}^{\text{i}}q_{y}^{\text{i}}\;}\text{cor(}Xq_{x}^{\text{i}},\;Yq_{y}^{\text{i}})$$

In CCA, the components **Xq_x_**^i^ and **Yq_y_**^i^ are called canonical variates and their correlations are the canonical correlations.

## Sparse canonical correlation analysis

The main limitation of applying CCA to omics data is that it requires an inversion of the correlation or covariance matrix [38, 49, 50], which cannot be calculated when the number of variables exceeds the number of observations [46]. In high-dimensional omics data where *p* ≫ *n*, application of these methods requires a regularization step. This may be accomplished by adding a ridge penalty, that is, adding a multiple of the identity matrix to the covariance matrix [51]. A sparse solution of the loading vectors (**Q_x_** and **Q_y_**) filters the number of variables and simplifies the interpretation of results. For this purpose, penalized CCA [52], sparse CCA [53], CCA-l1 [54], CCA elastic net (CCA-EN) [45] and CCA-group sparse [55] have been introduced and applied to the integrative analysis of two omics data sets. Witten *et al.* [36] provided an elegant comparison of various CCA extensions accompanied by a unified approach to compute both penalized CCA and sparse PCA. In addition, Witten and Tibshirani [54] extended sparse CCA into a supervised framework, which allows the integration of two data sets with a quantitative phenotype; for example, selecting variables from both genomics and transcriptomics data and linking them to drug sensitivity data.

### Partial least squares analysis

PLS is an efficient dimension reduction method in the analysis of high-dimensional omics data. PLS maximizes the covariance rather than the correlation between components, making it more resistant to outliers than CCA. Additionally, PLS does not suffer from the *p* ≫ *n* problem as CCA does. Nonetheless, a sparse solution is desired in some cases. For example, Le Cao *et al.* [56] proposed a sparse PLS method for the feature selection by introducing a LASSO penalty for the loading vectors. In a recent comparison, sPLS performed similarly to sparse CCA [45]. There are many implementations of PLS, which optimize different objective functions with different constraints, several of which are described by Boulesteix *et al.* [57].

### Co-Inertia analysis

CIA is a descriptive nonconstrained approach for coupling pairs of data matrices. It was originally proposed to link two ecological tables [13, 58], but has been successfully applied in integrative analysis of omics data [32, 43]. CIA is implemented and formulated under the duality diagram framework (Supplementary Information). CIA is performed in two steps: (i) application of a dimension reduction technique such as PCA, CA or NSCA to the initial data sets depending on the type of data (binary, categorical, discrete counts or continuous data) and (ii) constraining the projections of the orthogonal axes such that they are maximally covariant [43, 58]. CIA does not require an inversion step of the correlation or covariance matrix; thus, it can be applied to high-dimensional genomics data without regularization or penalization.

Though closely related to CCA [49], CIA maximizes the squared covariance between the linear combination of the preprocessed matrix, that is,

(11) $$\begin{array}{l} \text{for the}i\text{th dimension}:\mathrm{argma}x_{q_{x}^{\text{i}}q_{y}^{\text{i}}}\mathrm{cov}{\;}^{2}(Xq_{x}^{\text{i}},Yq_{y}^{\text{i}}). \end{array}$$

Equation (11) can be decomposed as:

(12) $${\text{cov}}^{2}\left({\text{Xq}}_{\text{x}}{}^{\text{i}},{\text{Yq}}_{\text{y}}{}^{\text{i}}\right)={\text{cor}}^{2}\left({\text{Xq}}_{\text{x}}{}^{\text{i}},{\text{Yq}}_{\text{y}}{}^{\text{i}}\right)\cdot \text{var}\left({\text{Xq}}_{\text{x}}{}^{\text{i}}\right)\cdot \text{var}\left({\text{Xq}}_{\text{y}}{}^{\text{i}}\right)$$

CIA decomposition of covariance maximizes the variance and the correlation between matrices and, thus is less sensitive to outliers. The relationship between CIA, Procrustes analysis [13] and CCA have been well described [49]. A comparison between sCCA (with elastic net penalty), sPLS and CIA is provided by Le Cao *et al.* [45]. In summary, CIA and sPLS both maximize the covariance between eigenvectors and efficiently identify joint and individual variance in paired data. In contrast, CCA-EN maximizes the correlation between eigenvectors and will discover effects present in both data sets, but may fail to discover strong individual effects [45]. Both sCCA and sPLS are sparse methods that select similar subsets of variables, whereas CIA does not include a feature selection step; thus, in terms of feature selection, results of CIA are more likely to contain redundant information in comparison with sparse methods [45].

Similar to classical dimension reduction approaches, the number of dimensions to be examined needs to be considered and can be visualized using a scree plot (similar to Figure 1D). Components may be evaluated by their associated variance [25], the elbow test or Q^2^ statistics, as described previously. For example, the Q^2^ statistic was applied to select the number of dimensions in the predictive mode of PLS [56]. In addition, when a sparse factor is introduced in the loading vectors, cross-validation approaches may be used to determine the number of variables selected from each pair of components. Selection of the number of components and optimization of these parameters is still an open research question and is an active area of research.

## Integrative analysis of multi-assay data

There is a growing need to integrate more than two data sets in genomics. Generalizations of dimension reduction methods to three or more data sets are sometimes called *K*-table methods [59–61], and a number of them have been applied to multi-assay data (Table 4). Simultaneous decomposition and integration of multiple matrices is more complex than an analysis of a single data set or paired data because each data set may have different numbers of variables, scales or internal structure and thus have different variance. This might produce global scores that are dominated by one or a few data sets. Therefore, data are preprocessed before decomposition. Preprocessing is often performed on two levels. On the variable levels, variables are often centered and normalized so that their sum of squared values or variance equals 1. This procedure enables all the variables to have equal contribution to the total inertia (sum of squares of all elements) of a data set. However, the number of variables may vary between data sets, or filtering/preprocessing steps may generate data sets that have a higher variance contribution to the final result. Therefore, a data set level normalization is also required. In the simplest *K*-table analysis, all matrices have equal weights. More commonly, data sets are weighted by their expected contribution or expected data quality, for example, by the square root of their total inertia or by the square root of the numbers of columns of each data set [62]. Alternatively, greater weights can be given to smaller or less redundant matrices, matrices that have more stable predictive information or those that share more information with other matrices. Such weighting approaches are implemented in multiple-factor analysis (MFA), principal covariates regression [63] and STATIS.

The simplest multi-omics data integration is when the data sets have the same variables and observations, that is, matched rows and matched columns. In genomics, these could be produced when variables from different multi-assay data sets are mapped to a common set of genomic coordinates or gene identifiers, thus generating data sets with matched variables and matched observations. Alternatively, a repeated analysis, or longitudinal analysis of the same samples and the same variables, could produce such data (one should note that these dimension reduction approaches do not model the time correlation between different datasets). There is a history of such analyses in ecology where counts of species and environment variables are measured over different seasons [49, 64, 65] and in psychology where different standardized tests are measured multiple times on study populations [66, 67]. Analysis of such *variables x samples x time* data are called a three-mode decomposition, triadic, cube or three-way table analysis, tensor decomposition, three-way PCA, three-mode PCA, three-mode Factor Analysis, Tucker-3 model, Tucker3, TUCKALS3, multi-block analysis, among others (Table 4). The relationship between various tensor or higher decompositions for multi-block analysis are reviewed by Kolda and Bader [68].

More frequently, we need to find associations in multi-assay data that have matched observations but have different and unmatched numbers of variables. For example, TCGA generated miRNA and mRNA transcriptome (RNAseq, microarray), DNA copy number, DNA mutation, DNA methylation and proteomics molecular profiles on each tumor. The NCI-60 and the Cancer Cell Line Encyclopedia projects have measured pharmacological compound profiles in addition to exome sequencing and transcriptomic profiles. Methods that can be applied to EDA of *K*-table of multi-assay data with different variables include MCIA, MFA, Generalized CCA (GCCA) and Consensus PCA (CPCA).

The *K*-table methods can be generally expressed by the following model:

(13) $$\begin{array}{c} X_{1}\;=\;FQ_{1}^{T}\;+\;E_{1}\\ \vdots \\ X_{k}\;=\;FQ_{k}^{T}\;+\;E_{k}\\ \vdots \\ X_{K}\;=\;FQ_{K}^{T}\;+\;E_{K} \end{array}$$

where there are *K* matrices or omics data sets **X_1_,…,X_K_**. For convenience, we assume that the rows of **X_k_** share a common set of observations but the columns of **X_k_** may each have different variables. **F** is the ‘global score' matrix. Its columns are the PCs and are interpreted similarly to PCs from a PCA of a single data set. The global score matrix, which is identical in all decompositions, integrates information from all data sets. Therefore, it is not specific to any single data set, rather it represents the common pattern defined by all data sets. The matrices **Q_k_**, with k ranging from 1 to *K*, are the loadings or coefficient matrices. A high positive value indicates a strong positive contribution of the corresponding variable to the ‘global score'. While the above methods are formulated for multiple data sets with different variables but the same observations, most can be similarly formulated for multiple data sets with the same variables but different observations [69].

### Multiple co-inertia analysis

MCIA is an extension of CIA which aims to analyze multiple matrices through optimizing a covariance criterion [60, 70]. MCIA simultaneously projects *K* data sets into the same dimensional space. Instead of maximizing the covariance between scores from two data sets as in CIA, the optimization problem used by MCIA is as following:

(14) $$argmax_{q_{1}^{i}..q_{k}^{i}..q_{K}^{i}}\sum_{k=1}^{K}cov^{2}(X_{k}^{i}q_{k}^{i},X_{}^{i}q_{}^{i})$$

for dimension *i* with the constraints that ||**q**_k_*^i^*|| = var (**X***^i^***q***^i^*) = 1, where **X** = (**X_1_**|…|**X_k_**|…|**X_K_**) and **q***^i^* holds the corresponding loading values (‘global' loading vector) [60, 69, 70].

MCIA derives a set of ‘block scores' **X**_k_*^i^***q**_k_*^i ^*using linear combinations of the original variables from each individual matrix. The global score **X***^i^***q***^i^* is then further defined as the linear combination of ‘block scores'. In practice, the global scores represent a correlated structure defined by multiple data sets, whereas the concordance and discrepancy between these data sets may be revealed by the block scores (for detail see ‘Example case study' section). MCIA may be calculated with the *ad hoc* extension of the NIPALS PCA [71]. This algorithm starts with an initialization step in which the global scores and the block loadings for the first dimension are computed. The residual matrices are calculated in an iterative step by removing the variance induced by the variable loadings (the ‘deflation' step). For higher order solutions, the same procedure is applied to the residual matrices and re-iterated until the desired number of dimensions is reached. Therefore, the computation time strongly depends on the number of desired dimensions. MCIA is implemented in the R package omicade4 and has been applied to the integrative analysis of transcriptomic and proteomic data sets from the NCI-60 cell lines [60].

## Generalized canonical correlation analysis

GCCA [71] is a generalization of CCA to *K*-table analysis [73–75]. It has also been applied to the analysis of omics data [36, 76]. Typically, MCIA and GCCA will produce similar results (for a more detailed comparison see [60]). GCCA uses a different deflation strategy than MCIA: it calculates the residual matrices by removing the variance with respect to the ‘block scores' (instead of ‘variable loadings' used by MCIA or ‘global scores' used by CPCA; see later). When applied to omics data where *p* ≫ *n*, a variable selection step is often integrated within the GCCA approach, which cannot be done in case of MCIA. In addition, as block scores are better representations of a single data set (in contrast to the global score), GCCA is more likely to find common variables across data sets. Witten and Tibshirani [54] applied sparse multiple CCA to analyze gene expression and Copy Number Variation (CNV) data from diffuse large B-cell lymphoma patients and successfully identified ‘*cis* interactions' that are both up-regulated in CNV and mRNA data.

### Consensus PCA

CPCA is closely related to GCCA and MCIA, but has had less exposure to the omics data community. CPCA optimizes the same criterion as GCCA and MCIA and is subject to the same constraints as MCIA [71]. The deflation step of CPCA relies on the ‘global score'. As a result, it guarantees the orthogonality of the global score only and tends to find common patterns in the data sets. This characteristic makes it is more suitable for the discovery of joint patterns in multiple data sets, such as the joint clustering problem.

### Regularized generalized canonical correlation analysis

Recently, Tenenhaus and Tenenhaus [69, 74] proposed regularized generalized CCA (RGCCA), which provides a unified framework for different *K*-table multivariate methods. The RGCCA model introduces some extra parameters, particularly a shrinkage parameter and a linkage parameter. The linkage parameter is defined so that the connection between matrices may be customized. The shrinkage parameter ranges from 0 to 1. Setting this parameter to 0 will force the correlation criterion (criterion used by GCCA), whereas a shrinkage parameter of 1 will apply the covariance criterion (used by MCIA and CPCA). A value between 0 and 1 leads to a compromise between the two options. In practice, the correlation criterion is better in explaining the correlated structure across data sets, while discarding the variance within each individual data set. The introduction of the extra parameters make RGCCA highly versatile. GCCA, CIA and CPCA can be described as special cases of RGCCA (see [69] and Supplementary Information). In addition, RGCCA also integrates a feature selection procedure, named sparse GCCA (SGCCA). Tenenhaus *et al.* [76] applied SGCCA to combine gene expression, comparative genomic hybridization and a qualitative phenotype measured on a set of 53 children with glioma. Sparse multiple CCA [54] and SGCCA [76] are available in the R packages PMA and RGCCA, respectively. Similarly, a higher order implementation of spare PLS is described in Zhao *et al.* [77].

### Joint NMF

NMF has also been extended to jointly factorize multiple matrices. In joint NMF, the values in the global score **F** and in the coefficient matrices (**Q_1_**,…,**Q_K_**) are nonnegative and there is no explicit definition of the block loadings. An optimization algorithm is applied to minimize an objective function, typically the sum of squared errors, i.e. $\sum_{\text{k}\;=\;1}^{K}{\text{E}}_{k}{}^{2}$. This approach can be considered to be a nonnegative implementation of PARAFAC, although it has also been implemented using the Tucker model [78–80]. Zhang *et al.* [81] apply joint NMF to a three-way analysis of DNA methylation, gene expression and miRNA expression data to identify modules in each of these regulatory layers that are associated with each other.

## Advantages of dimension reduction when integrating multi-assay data

**Figure 2.**
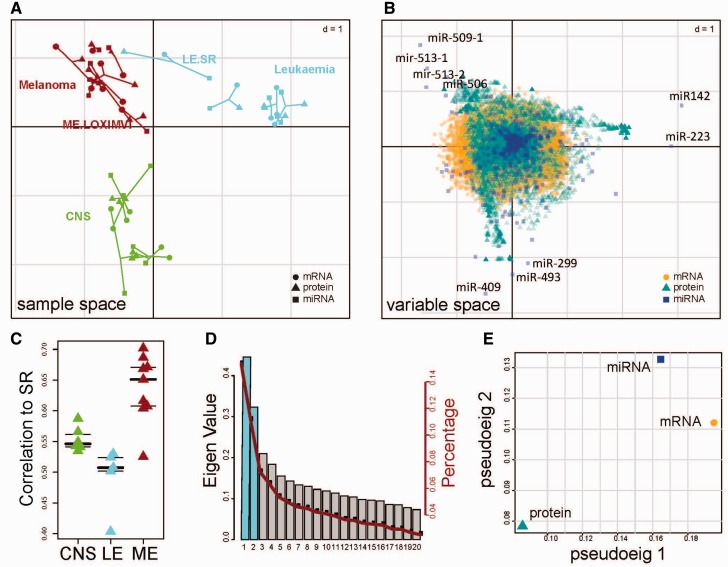
MCIA of mRNA, miRNA and proteomics profiles of melanoma (ME), leukemia (LE) and central nervous system (CNS) cell lines. (**A**) shows a plot of the first two components in sample space (sample ‘type' is coded by the point shape; circles for mRNAs, triangles for proteins and squares for miRNAs). Each sample (cell line) is represented by a “star”, where the three omics data for each cell line are connected by lines to a center point, which is the global score (**F**) for that cell line, the shorter the line, the higher the level of concordance between the data types and the global structure. (**B**) shows the variable space of MCIA. A variable that is highly expressed in a cell line will be projected with a high weight (far from the origin) in the direction of that cell line. Some miRNAs with a large distance from the origin are labeled, as these miRNAs are the strongly associated with cancer tissue of origin. (**C**) shows the correlation coefficients of the proteome profiling of SR with other cell lines. The proteome profiling of SR cell line is more correlated with melanoma cell line. There may be a technical issue with the LE.SR proteomics data. (**D**) A scree plot of the eigenvalues and (**E**) a plot of data weighting space. A colour version of this figure is available online at BIB online: http://bib.oxfordjournals.org.

Dimension reduction or latent variable approaches provide EDA, integrate multi-assay data, highlight global correlations across data sets, and discover outliers or batch effects in individual data sets. Dimension reduction approaches also facilitate down-stream analysis of both observations and variables (genes). Compared with cluster analysis of individual data sets, cluster analysis of the global score matrix (**F** matrix) is robust, as it aggregates observations across data sets and is less likely to reflect a technical or batch effect of a single data set. Similarly, dimension reduction of multi-assay data facilities downstream gene set, pathway and network analysis of variables. MCIA transforms variables from each data set onto the same scale, and their loadings (**Q** matrix) rank the variables by their contribution to the global data structure (Figure 2). Meng *et al.* report that pathway or gene set enrichment analysis (GSEA) of the transformed variables is more sensitive than GSEA of each individual data set. This is both because of the re-weighting and transformation of variables, but also because GSEA on the combined data has greater coverage of variables (genes) thus compensating for missing or unreliable information in any single data set. For example, in Figure 2B, we integrate and transform mRNA, proteomics and miRNA data on the same scale, allowing us to extract and study the union of all variables.

## Example case study

To demonstrate the integration of multi-data sets using dimensions reduction, we applied MCIA to analyze mRNA, miRNA and proteomics expression profiles of melanoma, leukemia and CNS cells lines from the NCI-60 panel. The graphical output from this analysis, a plot of the sample space, variable space and data weighting space are provided in Figure 2A, B and E. The eigenvalues can be interpreted similarly to PCA, a higher eigenvalue contributes more information to the global score. As with PCA, researchers may be subjective in their selection of the number of components [24]. The scree plot in Figure 2D shows the eigenvalues of each global score. In this case, the first two eigenvalues were significantly larger, so we visualized the cell lines and variables on PC1 and PC2.

In the observation space (Figure 2A), assay data (mRNA, miRNA and proteomics) are distinguished by shape. The coordinates of each cell line (**F**_k_ in Figure 2A) are connected by lines to the global scores (**F**). Short lines between points and cell line global scores reflect high concordance in cell line data. Most cell lines have concordant information between data sets (mRNA, miRNA, protein) as indicated by relatively short lines. In addition, the RV coefficient [82, 83], which is a generalized Pearson correlation coefficient for matrices, may be used to estimate the correlation between two transformed data sets. The RV coefficient has values between 0 and 1, where a higher value indicates higher co-structure. In this example, we observed relatively high RV coefficients between the three data sets, ranging from 0.78 to 0.84. It was recently reported that the RV coefficient is biased toward large data sets, and a modified RV coefficient has been proposed [84].

In this analysis (Figure 2A), cell lines originating from the same anatomical source are projected close to each other and converge in clusters. The first PC separates the leukemia cell lines (positive end of PC1) from the other two cell lines (negative end of PC1), and PC2 separates the melanoma and CNS cell lines. The melanoma cell line LOX-IMVI, which lacks the melanogenesis, is projected close to the origin, away from the melanoma cluster. We were surprised to see that the proteomics profile of leukemia cell line SR was projected closer to melanoma rather than leukemia cell lines. We examined within tumor type correlations to the SR cell line (Figure 2C). We observed that the SR proteomics data had higher correlation with melanoma compared with to leukemia cell lines. Given that the mRNA and miRNA profiles of LE_SR are closer to the leukemia cell lines, it suggests that there may have been a technical error in generating the proteomics data on the SR cell line (Figure 2A and C).

MCIA projects all variables into the same space. The variable space (**Q_1_**,…, **Q_K_**) is visualized in Figure 2B. Variables and samples projected in the same direction are associated. This allows one to select the variables most strongly associated with specific observations from each data set for subsequent analysis. In our previous study [85], we have shown that the genes and proteins highly weighted on the melanoma side (positive end of second dimension) are enriched with melanogenesis functions, and genes/proteins highly weighted on the protein side are highly enriched in T-cell or immune-related functions.

We examined the miRNA data to extract the miRNAs with the most extreme weights on the first two dimensions. miR-142 and miR-223, which are active and expressed in leukemia [82, 83, 86–88], had high weights on the positive end of both the first and second axis (close to the leukemia cell lines sample space, Figure 2A). miR-142 plays an essential role in T-lymphocyte development. miR-223 is regulated by the Notch and NF-kB signaling pathways in T-cell acute lymphoblastic leukemia [89].

The miRNA with strongest association to CNS cell lines was miR-409. This miRNA is reported to promote the epithelial-to-mesenchymal transition in prostate cancer [90]. In the NCI-60 cell line data, CNS cell lines are characterized more by a pronounced mesenchymal phenotype, which is consistent with high expression of this miRNA. On the positive end of the second axis and negative end of the first axis (which corresponds to melanoma cell lines in the sample space, Figure 2A), we found miR-509, miR-513 and miR-506 strongly associated with melanoma cell lines, which are reported to initiate melanocyte transformation and promote melanoma growth [85].

## Challenges in integrative data analysis

EDA is widely used and well accepted in the analysis of single omics data sets, but there is an increasing need for methods that integrate multi-omics data, particularly in cancer research. Recently, 20 leading scientists were invited to a meeting organized by *Nature Medicine*, *Nature Biotechnology* and the Volkswagen Foundation. The meeting identified the need to simultaneously characterize DNA sequence, epigenome, transcriptome, protein, metabolites and infiltrating immune cells in both the tumor and the stroma [91]. The TCGA pan-cancer project plans to comprehensively interrogate multi-omics data across 33 human cancers [92]. The data are biologically complex. In addition to tumor heterogeneity [91] there may be technical issues, batch effects and outliers. EDA approaches for complex multi-omics data are needed.

We describe emerging applications of multivariate approaches to omics data analysis. These are descriptive approaches that do not test a hypothesis or generate a *P*-value. They are not optimized for variable or biomarker discovery, although the introduction of sparsity in variable loadings may help in the selection of variables for downstream analysis. Few comparisons of different methods exist, and the numbers of components and the sparsity level have to be optimized. Cross-validation approaches are potentially useful but this still remains an open area of research.

Another limitation of these methods is that, although variables may vary among data sets, the observations need to be matchable. Therefore, researchers need to have careful experimental design in the early stage of a study. There is an extension of CIA for the analysis of unmatched samples [93], which combines a Hungarian algorithm with CIA to iteratively pair samples that are similar but not matched. Multi-block and multi-group methods (data sets with matched variables) have been reviewed recently by Tenenhaus and Tenenhaus [69].

The number of variables in genomics data is a challenge to traditional EDA visualization tools. Most visualization approaches were designed for data sets with fewer variables. Within R, new packages including ggord are being developed. Dynamic data visualization is possible using ggvis, plotly, explor and other packages. However, interpretation of long lists of biological variables (genes, proteins, miRNAs) is challenging. One way to gain more insight into lists of omics variables is to perform a network, gene set enrichment or pathway analysis [94]. An attractive feature of decomposition methods is that variable annotation, such as Gene Ontology or Reactome, can be projected into the same space, to determine a score for each gene set (or pathway) in that space [32, 33, 60].

## Conclusion

Dimension reduction methods have a long history. Many similar methods have been developed in parallel by multiple fields. In this review, we provided an overview of dimension reduction techniques that are both well-established and maybe new to the multi-omics data community. We reviewed methods for single-table, two-table and multi-table analysis. There are significant challenges in extracting biologically and clinically actionable results from multi-omics data, however, the field may leverage the varied and rich resource of dimension reduction approaches that other disciplines have developed.

Key Points

- There are many dimension-reduction methods, which can be applied to exploratory data analysis of a single data set, or integrated analysis of a pair or multiple data sets. In addition to exploratory analysis, these can be extended to clustering, supervised and discriminant analysis.
- The goal of dimension reduction is to map data onto a new set of variables so that most of the variance (or information) in the data is explained by a few new (latent) variables.
- Multi-data set methods such as multiple co-inertia analysis (MCIA), multiple factor analysis (MFA) or canonical correlations analysis (CCA) identify correlated structure between data sets with matched observations (samples). Each data set may have different variables (genes, proteins, miRNA, mutations, drug response, etc).
- MCIA, MFA, CCA and related methods provide a visualization of consensus and incongruence in and between data sets, enabling discovery of potential outliers, batch effects or technical errors.
- Multi-dataset methods transform diverse variables from each data set onto the same space and scale, facilitating integrative variable selection, gene set analysis, pathway and downstream analyses.

## R Supplement

R code to re-generate all figures in this article is available as a Supplementary File. Data and code are also available on the github repository https://github.com/aedin/NCI60Example.

## Supplementary Data

Supplementary data are available online at http://bib.oxfordjournals.org/.

Supplementary Data
