# Supplementary material for: Dimension reduction techniques for the integrative analysis of multi-omics data
Source: Brief Bioinform. 2016 Mar 11;17(4):628–41. doi: 10.1093/bib/bbv108 (PMC4945831; doi:10.1093/bib/bbv108)
Supplement: Supplementary Data [file supp_17_4_628__index.html]

Dimension reduction techniques for the integrative analysis of multi-omics data — Dimension reduction techniques for the integrative analysis of multi-omics data — Supplementary Data 

# Dimension reduction techniques for the integrative analysis of multi-omics data

## Supplementary Data

files

- Supplementary Data - docx file
