# Supplementary material for: Dimension reduction techniques for the integrative analysis of multi-omics data
Source: Brief Bioinform. 2016 Mar 11;17(4):628–41. doi: 10.1093/bib/bbv108 (PMC4945831; doi:10.1093/bib/bbv108)
Supplement: Supplementary Data [file supp_bbv108_Supplemental_Information.docx]

**Supplemental Information**

**NIPALS Algorithm**

| *Algorithm 1 – Nonlinear Iterative Partial Least square (NIPALS) algorithm for PCA.* |
| --- |
| initialize **X_0_** = **X**  for i = 1, ..., r  initialize **f^1^** as the first column in **X_i-1_**  1. **q^i^**=**X_i-1_ f^i^**/(**f^iT^f^i^**)  2. **q^i^** = **q^i^**/(**q^iT^q^i^**)^1/2^  3. **f^i^**= **X_i-1_ q^i^**  4. Check convergence of **f^i^** and **q^i^**, if not, go back to step 1; otherwise step 5.  5. **X_i_** = **X_i-1_** - **f^i^q^i^**^T^ # deflation step |

This algorithm is particularly useful in the analysis of omics data because, when compared to SVD, it requires less computation time and can handle missing data. The NIPALS algorithm calculating components and loadings via regression for each dimension (step 1 to 3), hence a small number of missing values will not affect the procedure. For a higher order solution, the same procedure is applied on the residual matrix calculated from the deflation step (step 5 in algorithm 1). The residual matrix can be viewed as the regression of **X^i-1^** onto **f^i^** or removing the variance explained by **f^i^** from **X^i-1^.** Therefore, it is faster when the matrix **X** is large because it calculates a subset of PCs whereas SVD computes all PCs. Of particular interest in multi-omics data analysis, this algorithm may be generalized to discover the correlated structure in more than one datasets (see sections on the analysis of multi omics datasets).

**NMF**

Non-negative matrix factorization (NMF) is an approach adopted from signal processing where it was used to solve the blind source deconvolution problem and has been widely applied in clustering analysis, face recognition and text mining [1]. NMF is also called self-modeling curve resolution or positive matrix factorization. NMF can be defined by the following model:

| **X** = **WH**+**E**, subject to the constraint **W**, **H** >= 0. | (9) |
| --- | --- |

**W** and **H** are *n×r* and *r×p* matrices, respectively; **E** is an error term accounting for inaccurate reconstruction. Similar to PCA and other decomposition approaches, NMF seeks to explain the principal sources of variance in a dataset using a small number (*r*) of vectors. But NMF solves this problem by minimizing the sum of squared errors **E**. Furthermore, it forces a positive or non-negative constraint on the resulting data matrices and [similar to Independent Component Analysis; 2] does not require orthogonality or independence in the components. This allows NMF to identify overlapping patterns in components. At the same time, the non-negative constraint guarantees that only the additive combinations of latent variables are allowed, which has more intuitive meaning since many biological variables could only be represented by positive values, such as protein concentration and count data.

**The duality diagram**

In the 1970s, French statisticians Cazes [3], Cailliez and Pages [4] developed a unifying framework, called the duality diagram, which provides an elegant approach to formulate dimension reduction methods in a similar way [see also 5, 6-8]. This framework is based on the statistical triplet (**X, R, D**) where **X** is a matrix with *n* rows (observations or samples) and *p* columns (variables or genes). The row-column duality suggested that rows and columns can be viewed in a symmetrical way, i.e. the matrix could be viewed as consisting of *n* points (variables/genes) defined in the space ℝ^p^; or *p* points (samples) defined in the space ℝ^n^.

**R** and **D** are positive symmetric matrices with dimension *p×p* and *n×n*, which provide a metric used to compute inner products in ℝ^p^ and ℝ^n^, respectively. From a geometrical point of view, analyzing the statistical triplet (**X, R, D**) can be formulated as either finding principal axes of a dataset containing *n* points in ℝ^p^ or as finding the principal components of *p* points in ℝ^n^. The introduction of **R** and **D** makes the duality diagram a highly versatile framework that includes many dimension reduction methods as special cases. For example, PCA in the original scale can be formulated as a duality diagram with **R = I** (identity) and **D** is a diagonal matrix consisting of uniform row weights (1/*n*). CA can be formulated as a duality diagram by defining **R** and **D** as the marginal frequencies of the original matrix and by standardizing **X** so that it captures the departure from independence of the original data. If only **R** or **D** are defined as above, it formulates the NSCA. Co-inertia analysis or decomposition of multiple matrices is a simple extension of this. Further examples and mathematical details are available in several excellent reviews [7-9]. Dimension reduction approaches based on the duality diagram are implemented in the R package ade4 [10].

**Co-Inertia Analysis**

CIA is a descriptive non-constrained approach for coupling pairs of data matrices. It was originally proposed to link two ecological tables [9, 11], but it has already been successfully applied in omics data analysis [12, 13]. CIA is implemented under the duality diagram framework in the ade4 package. In this scheme, CIA analyzes two statistical triplets (**X**,**L**,**D**) and (**Y**,**R**,**D**). In physics, the inertia of a set of points relative to a reference point is defined by the weighted sum of squared distances between each considered point and the reference point. Correspondingly, the inertia of a centered matrix (mean is equal to zero) is simply the sum of the squared matrix elements. The inertia of the matrix **X** defined by the metrics **L** and **D** is the weighted sum of its squared values, that is:

| trace(**XLX**^T^**D**). | (1) |
| --- | --- |

The inertia equals the total variance of **X** when **X** is centered, **L** is the Euclidean metric and **D** is a diagonal matrix with *l_i_* = 1/*n*. However, the concept of inertia is more flexible since different metrics may be used to account for different types of data. For example, if **L** and **D** are defined according to CA the inertia of **X** is proportional to the χ^2^ statistics.

When coupling a pair of datasets, the co-inertia between two matrices, **X** and **Y**, is calculated as

| trace(**XLX**^T^**DYRY**^T^**D**). | (2) |
| --- | --- |

CIA decomposes the co-inertia criteria into a set of orthogonal axes.

**Regularized generalized CCA**

Recently, Tenenhaus et al. proposed regularized generalized CCA, which provides a unified framework for different multi-table multivariate methods [15]. This method was defined as the following problem:

| $\mathrm{argmax}_{q_{1},...q_{K}}\sum_{k,j=1,k\neq j}^{K} c_{kj}g(\mathrm{cov}(\mathbf{X}_{\mathbf{k}}\mathbf{q}_{\mathbf{k}},\mathbf{X}_{\mathbf{j}}\mathbf{q}_{\mathbf{j}}))$  subject to the constraint $\tau_{k}\left\Vert\mathbf{q}_{\mathbf{k}}\right\Vert^{2}+(1-\tau_{k})\mathrm{var}(\mathbf{X}_{\mathbf{k}}\mathbf{q}_{\mathbf{k}})=1$ | (17) |
| --- | --- |

where *c_kj_* is a linkage function so that c_kj_ = 1 if data k and j are connected and otherwise c_kj_ = 0. The function *g* represents different optimization criteria; it could be an identity (i.e. sum of covariance criterion), a square function (i.e. sum of squared covariance criterion) or an absolute function (i.e. sum of the absolute covariance criterion). Last, τ is a shrinkage parameter ranging from 0 to 1; setting τ to 0 will force the "canonical variates" or "components" to unit variance ($\mathrm{var}(\mathbf{X}_{\mathbf{k}}\mathbf{q}_{\mathbf{k}})=1$), in which case the covariance is equal to the correlation (i.e. criterion used by GCCA). As described before (equation (15)), the correlation criterion is better in explaining the correlated structure across datasets, thus discarding the variance within each individual dataset. Setting τ equal to 1 will normalize the loadings (q_k_) to 1, which applies the covariance criterion. A value between 0 and 1 will lead to a compromise between the two options. The introduction of the above three parameters (*c,g,*τ) enables RGCCA to perform many similar methods, including GCCA, CIA and CPCA, as particular cases. In MCIA and CPCA, the squared covariance between the individual datasets are compared to the global structure. Therefore, it is comparable with RGCCA when **X_j_** is the concatenated matrix and *g* is the squared function (equation 17). In this context, GCCA can be expressed as a mixed correlation and covariance criteria [15].

**R Code to generate figures**

R code to generate figures is available as a supplement. An rmarkdown version of this, image files of the results and the data necessary to generate the figures is available on the github repository <https://github.com/aedin/NCI60Example>. To install this package from github, type the following in R.

*library(devtools)*

*install_github("aedin/NCI60Example")*

*library(NCI60Example)*

*data(nci60)*

1. Lee DD, Seung HS. Learning the parts of objects by non-negative matrix factorization. Nature. 1999;401(6755):788-91. doi: 10.1038/44565. PubMed PMID: 10548103.

2. Comon P. Independent component analysis, a new concept? Signal processing. 1994.

3. Cazes P. Application de l’analyse des donn´ees au traitement de problemes geologiques. 1970.

4. F Cailliez JP. Introduction à l’analyse des données. 1976.

5. Thioulouse J. Simultaneous analysis of a sequence of paired ecological tables: A comparison of several methods. The Annals of Applied Statistics. 2011. doi: 10.2307/23069331.

6. Dray S. Analysing a Pair of Tables: Coinertia Analysis and Duality Diagrams. Visualization and Verbalization of Data. 2014.

7. De la Cruz O, Holmes S. The Duality Diagram in Data Analysis: Examples of Modern Applications. Ann Appl Stat. 2011;5(4):2266-77. PubMed PMID: 22282721; PubMed Central PMCID: PMCPMC3265363.

8. Escoufier Y. The duality diagram: a means for better practical applications. Develoments in Numerical Ecology. 1987. doi: 10.1007/978-3-642-70880-0_3.

9. Dray S, Chessel D, Thioulouse J. Co-inertia analysis and the linking of ecological data tables. Ecology. 2003.

10. Dray S, Dufour AB. The ade4 package: implementing the duality diagram for ecologists. Journal of statistical software. 2007.

11. Dolédec S, Chessel D. Co‐inertia analysis: an alternative method for studying species–environment relationships. Freshwater biology. 1994. doi: 10.1111/j.1365-2427.1994.tb01741.x.

12. Culhane AC, Perriere G, Higgins DG. Cross-platform comparison and visualisation of gene expression data using co-inertia analysis. BMC Bioinformatics. 2003;4:59. doi: 10.1186/1471-2105-4-59. PubMed PMID: 14633289; PubMed Central PMCID: PMCPMC317282.

13. Fagan A, Culhane AC, Higgins DG. A multivariate analysis approach to the integration of proteomic and gene expression data. Proteomics. 2007;7(13):2162-71. doi: 10.1002/pmic.200600898. PubMed PMID: 17549791.

14. Hanafi M, Kohler A, Qannari EM. Connections between multiple co-inertia analysis and consensus principal component analysis. Chemometrics and intelligent laboratory …. 2011.

15. Tenenhaus A, Tenenhaus M. Regularized generalized canonical correlation analysis. Psychometrika. 2011. doi: 10.1007/s11336-011-9206-8.
